# Graphene and water-based elastomers thin-film composites by dip-moulding

**DOI:** 10.1016/j.carbon.2016.05.032

**Published:** 2016-09

**Authors:** Maria Iliut, Claudio Silva, Scott Herrick, Mark McGlothlin, Aravind Vijayaraghavan

**Affiliations:** a School of Materials and National Graphene Institute, University of Manchester, Manchester M13 9PL, UK; b Department of Fundamental Chemistry, Institute of Chemistry, University of São Paulo, São Paulo, Brazil; c Apex Medical Technologies, San Diego, CA 92121, USA

## Abstract

Thin-film elastomers (elastic polymers) have a number of technologically significant applications ranging from sportswear to medical devices. In this work, we demonstrate that graphene can be used to reinforce 20 micron thin elastomer films, resulting in over 50% increase in elastic modulus at a very low loading of 0.1 wt%, while also increasing the elongation to failure. This loading is below the percolation threshold for electrical conductivity. We demonstrate composites with both graphene oxide and reduced graphene oxide, the reduction being undertaken in-situ or ex-situ using a biocompatible reducing agent in ascorbic acid. The ultrathin films were cast by dip moulding. The transparency of the elastomer films allows us to use optical microscopy image and confirm the uniform distribution as well as the conformation of the graphene flakes within the composite.

## 1 Introduction

Elastomers are viscoelastic polymers with weak inter-molecular forces that exist in an amorphous state above their glass transition temperature. Elastomers can be classified as thermosets or thermoplastics; thermosets such as various rubbers require cross-linking by a curing process such as vulcanisation, whereas thermoplastics such as polystyrene are not cross-linked [1], [2], [3] Elastomers enjoy a wide range of applications in everyday products such as tires, shoe-soles, gloves and prophylactics [4], [5], [6], [7] The most common thermoset elastomer is vulcanised natural rubber latex (NRL) which consists of the elastomer *cis*-1,4-polyisoprene together with a small fraction of proteins, fatty acids and other organic and inorganic compounds. Rubber is most commonly processed in the form of a latex, a stable dispersion (emulsion) of polymer microparticles in an aqueous medium [8] which is used in this study. Also common are thermoplastic polyurethanes (PU), which are linear segmented block copolymers composed of hard and soft segments [9]. While polyurethanes are generally not dispersed in water, there do exist water-based PUs such as the anionic aliphatic polyester polyurethane emulsion in water used in this study [10]. At ambient conditions, elastomers are soft materials with elastic modulus of 1–10 MPa and ultimate tensile strength of 10s of MPa, and can withstand elongations of 700%–1000%.

Elastomer products can be formed by a variety of techniques such as injection moulding, dip moulding, blow moulding, compression moulding, etc. There are a wide range of elastomer products which are produced worldwide predominantly by dip-moulding method. Such applications include gloves, plastic bags, condoms, coatings on metallic objects for corrosion protection, rubber grip coatings, etc. Dip-coating is used because it can form a thin, conformal coating over a range of arbitrary and complex shapes; this is not achieved by other moulding or coating methods. The coatings may either be left on the object (e.g. for corrosion or damage protection) or removed and utilised as a thin-film such as gloves and condoms.

For thin-film applications such as condoms and gloves, where the elastomers are formed to thicknesses of less than 100 microns, traditional fillers with particle sizes in the micro-scale or larger are unsuitable. Nano-scale fillers such as graphene [11], [12] are promising candidates to improve the elastic properties of elastomer thin-films; in the ideal case to improve the elastic modulus, ultimate tensile strength and strain to failure simultaneously. In this study we demonstrate that the addition of a small loading of graphene (in the form of graphene oxide (GO) [13] and reduced graphene oxide (rGO) [14], [15]) can achieve this ideal reinforcement. Graphene in its various forms (GO, rGO, graphene nanoplatelets, expanded graphite, etc.) have achieved improved mechanical, thermal and electrical properties in elastomers [12]. However, such composites have usually focussed on thick films with high graphene loading (>1 wt%) and sacrificing elasticity (ultimate elongation) for improved modulus.

## 2 Experimental

### 2.1 Sample preparation

#### 2.1.1 Graphene oxide

GO was produced by oxidising graphite according to a modified Hummers method [16], followed by exfoliation and purification. The GO was also further sorted to yield dispersions with well-defined flake size distribution and 100% yield of mono-atomic GO layers. Additional characterisation of the GO and rGO, such as Raman spectra and X-ray photoelectron spectroscopy (XPS) data, are shown in online supporting information.

#### 2.1.2 Elastomer blends

The wPU used in this work was Impranil^®^ DLN-SD (Covestro AG). The NRL used in this work was Revultex^®^ HR 8/012 (Revertex (Malaysia) SDN BHD). wPU at 40% solids or NRL at 60% solids content was blended with graphene oxide at 2.5 mg/ml using a magnetic stirrer at room temperature to obtain a homogenous blend to obtain the GO/wPU or GO/NRL blends. To obtain rGO, 1 M ascorbic acid [17] was added to the GO/wPU mix while continuously stirring at room temperature, followed by 25% ammonium hydroxide solution to maintain the pH of the solution between 9 and 10. The mixture was heated in a closed container to 75 °C while stirring for 48 h to obtain the rGO/wPU blend. In the case of NRL, the GO at 2.5 mg/ml was mixed with 1 M ascorbic acid and 25% ammonium hydroxide and heated in a closed container to 30 °C for 48 h to produce rGO in a charge-stabilised gel-like phase, which was then added to the NRL and blended using a magnetic stirrer at room temperature. The ratio of wPU and NRL to GO was tailored to yield final required graphene loading in the dried composite.

#### 2.1.3 Graphene/elastomer composites

**Fig. 1:**
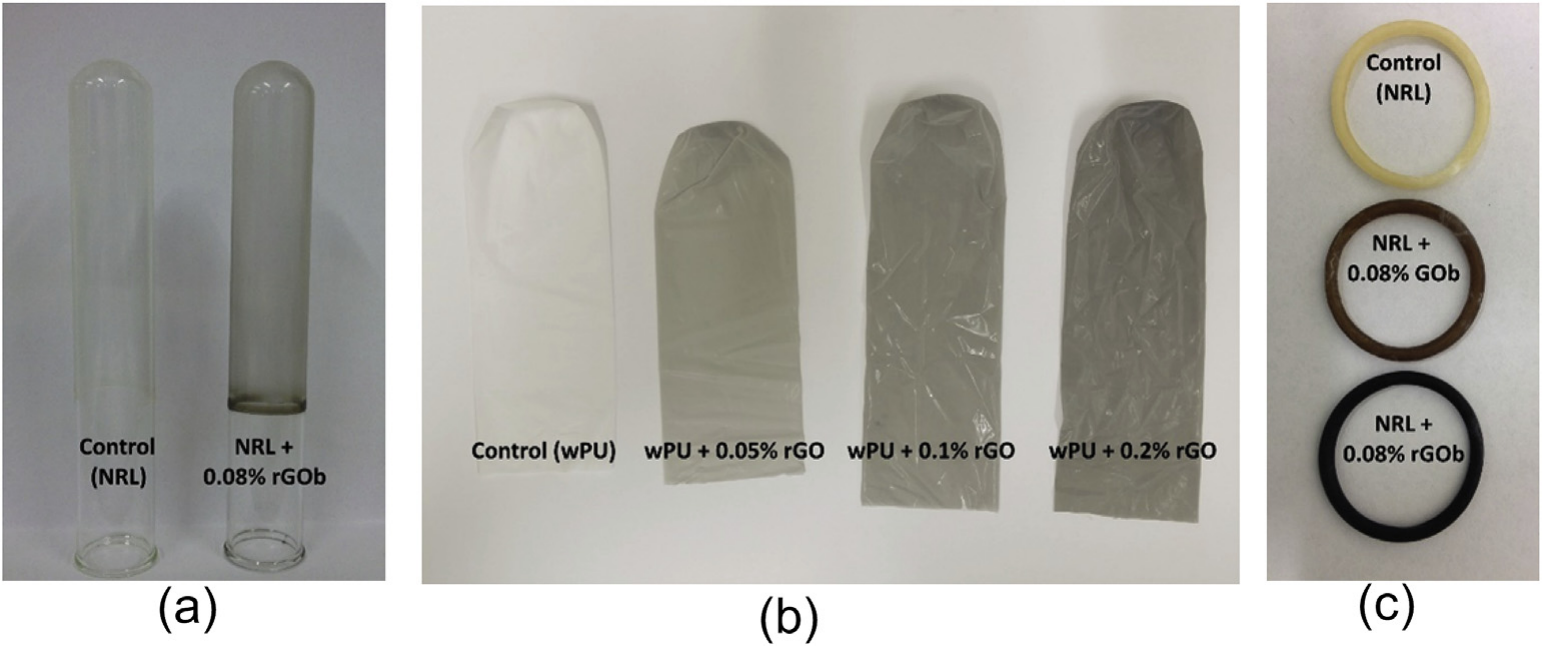
**a**. Photographs of NRL and rGO/NRL composite films after dip molding on glass mandrels. **b**. Photographs of wPU and rGO/wPU composites at different rGO loadings after release from their glass mandrels and **c**. NRL, GO/NRL and rGO/NRL ring tensile test specimens obtained from (a). (A colour version of this figure can be viewed online.)

wPU films were dipped on 32 mm OD smooth glass mandrels and NRL films were dipped on 22 mm OD smooth glass mandrels without any release agents or other processing aids, as shown in Fig. 1a. All drying was done in a forced air convection oven at 80 °C for 20 min to dry the water content out completely. Corn starch was applied as a slurry in water before removing the films from the mandrel to ensure all surfaces remained coated with corn starch at all times. The slurry aids in both release of the thin film from the mandrill and prevents the sticking of the film to itself after removal.

#### 2.1.4 Tensile testing

Tensile testing of graphene/wPU composite was performed followed the procedures in ASTM Standard D3492 – 97 [18] using flat-ring tensile test specimens (Fig. 1b). Tensile testing of graphene/NRL was performed following the procedures in ISO Standard 4072:2002 [19] using cylindrical-ring tensile test specimens, as shown in Fig. 1c. All values are the median of 6 test samples. In both cases, ultimate tensile strength (and elastic moduli) were calculated according to the formula T = F_b_/2A_x_, where: T = tensile strength, F_b_ = breaking force (or force at specific elongation for modulus values), and A_x_ is the cross-section area of the ring. The formula used for elongation at break is: E = 100((2D + G−C)/C), where: E = elongation at break, %, D = distance between centres of rollers at break, G = circumference of one roller, C = circumference of the specimen.

## 3 Results and discussion

**Fig. 2:**
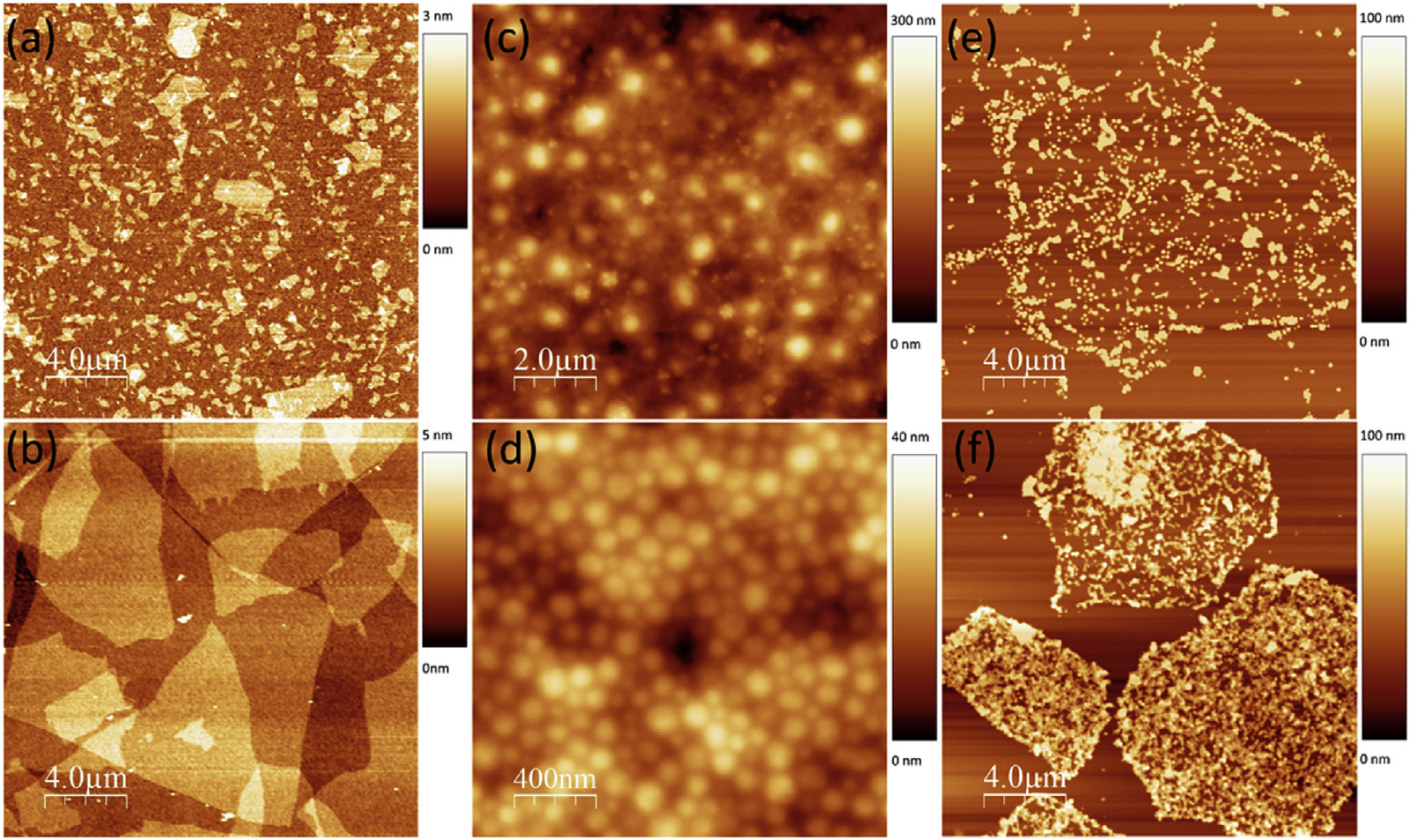
AFM images of **a**. small and **b**. large GO flakes; **c**. NRL and **d**. wPU particles and **e**. GO flakes covered with wPU and **f**. rGO flakes covered with wPU. (A colour version of this figure can be viewed online.)

We used two ‘grades’ of GO, with flake size distribution of <1 μm (small), and 3–30 μm (large). Fig. 2 (a–b) shows atomic force microscopy images of GO flakes, indicating flake size and thickness. Two types of elastomers were used for this work – natural rubber latex (NRL) and water-based polyurethane (wPU). These two materials are representative of two systems – thermoset and thermoplastic elastomers respectively. The NRL emulsion contains particles that are 100s of nanometres in size, whereas the wPU emulsion contains particles which are 10s of nanometres in size, as evidences by AFM (Fig. 2c and d). 4 types of graphene/wPU formulations were prepared: (1) Control – pure wPU, (2) 0.05 wt% rGO, (3) 0.1 wt% rGO and (4) 0.2 wt% rGO. 4 types of graphene/NRL formulations were prepared: (1) Control – pure NRL, (2) 0.08 wt% large GO, (3) 0.08 wt% large rGO and (4) 0.08 wt% small rGO.

Fig. 1 shows optical images of the graphene/NRL composite thin-films dip-coated on the glass mandrels, ring tensile test specimens of graphene/NRL rolled off the glass mandrels and graphene/wPU composite thin-films after released from the mandrel. Film thickness was measured using a snap gauge; the composite films were between 20 and 30 microns thick.

**Fig. 3:**
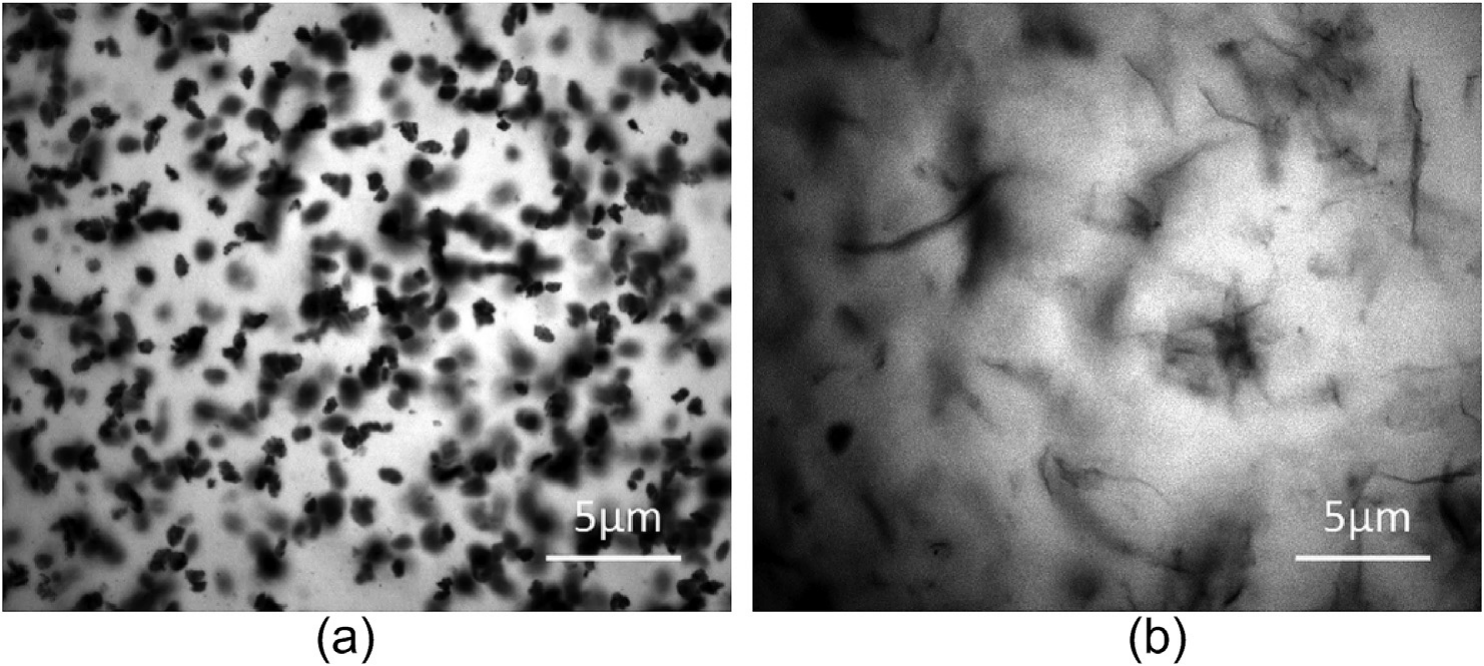
Optical micrographs of **a**. small and **b**. large rGO flakes in NRL matrix. (A colour version of this figure can be viewed online.)

Due to the good transparency of NRL thin-films, the distribution of rGO flakes inside the NRL matrix could be imaged by optical microscopy in reflectance mode (Fig. 3). In order to enhance the contrast of the rGO flakes in the NRL matrix, special optical microscopy samples were prepared by dip-coating rGO/NRL thin-films on a silicon substrate with a 300 nm SiO_2_ surface layer. Additional magnifications and images are shown in online supporting information. GO, on the other hand, did not offer sufficient optical contrast to be imaged optically in the NRL matrix. Excellent uniform dispersion of individual graphene flakes could be observed in the NRL matrix for rGO for small and large flakes. The NRL appears to stabilise the dispersion of the graphene effectively and prevent aggregation prior to or during the dip moulding process. The small rGO flakes were general flat, but occurring in small clusters. Large rGO flakes were generally individually dispersed, but not flat. This morphology of the different rGO flake sizes could correlate to the resulting mechanical properties described subsequently. This also confirms that the general assumption of graphene as a flat sheet [20], [21], [22] in a composite is not valid for larger graphene flakes; the persistence length of GO and rGO flakes at least in elastomer composites is on the order of 1 μm.

Since the wPU used here is an anionic aliphatic polyester and GO is also anionic [23], [24], [25] it can be expected that the interaction between GO and wPU is weak. This was confirmed by AFM imaging of individual GO and rGO flakes in the wPU blend after excess polymer was removed by washing and ultracentrifugation (Fig. 2 c–d). rGO flakes were observed to be covered entirely with wPU indicating a strong interaction, whereas GO flakes were only sparsely covered by wPU indicating a weak interaction. Consequently, only rGO was used in wPU blends.

Table 1, Table 2 summarises the results of mechanical testing of the graphene/wPU and graphene/NRL composite thin-films respectively. The corresponding load-extension curves are included in online supporting information. For wPU, we observe up to 65% increase in ultimate tensile strength and a 6% increase in ultimate elongation for 0.2 wt% loading. For any given elongation, we also observe that the tensile modulus increased by at least 35%; the results are reported in the tables in industry-standard format in steps of 100% elongation. For NRL, we observe a 35% increase in ultimate tensile strength accompanied by a 7% increase in the ultimate elongation for 0.08 wt% loading. In the case of the small rGO flakes, we observe a higher tensile modulus for equivalent elongation compared to large rGO, however, the small rGO flakes resulted in a reduced ultimate tensile strength and ultimate elongation.

This could be attributed to the morphology of the small and large graphene flakes within the elastomer matrix. The built-in undulations in the large graphene flakes compared to the flat small graphene flakes is attributed to the higher ultimate elongation whereas the same morphology combined with the clustering of small graphene flakes is attributed to the lower ultimate elongation. There are two major contributing factors to the different aggregation rates of small and large rGO flakes. Rheology measurements on similar concentration dispersions of small and large GO flakes have shown that larger flake dispersions have higher viscosities, especially at low shear rate and amplitudes, both in the GO dispersion [26] and when incorporated into a composite [27]. This indicates that smaller flakes are more mobile in a dispersion and therefore prone to faster aggregation. It was also observed that at the high concentration and high pH at which the flakes are added into the polymer, the large rGO flakes form a sterically stabilised gel phase, which preserves the isolation of the flakes prior to addition into the polymer dispersion, whereas small rGO dispersions remain fluid even at high concentrations and the aggregation commences prior to addition into the polymer dispersion. This second consideration can be overcome in the case of wPU by undertaking in-situ reduction as demonstrated here. The higher ultimate tensile strength obtained with large graphene particles can be attributed to more efficient load transfer between the matrix and the filler when the graphene particles have a larger than critical lateral size, as was previous demonstrated by Gong et al. [28]; in this work a critical lateral flake size of 2 μm was experimentally obtained for complete load transfer between a soft PMMA matrix and a rigid graphene flake. We expect that a similar critical length exists for the GO/elastomer systems studied here, and that the GO flake sizes used in this work fall entirely on either size of this critical length.

The above results, taken together, demonstrates that the addition of a small amount of optimum graphene filler to NRL and wPU significantly improves the mechanical properties of these elastomers in their thin-film form.

One of the various ways to test the integrity of elastomer thin-film products, such as gloves and condoms, is electrical conductance test. This is the only standard non-destructive test that is integrated into the production flow, where electrical conductivity across the elastomer thin-film is interpreted as a flaw such as a pin-hole leading to product rejection. To avoid invalidating this unique industrial standard test, it is essential that the addition of a conductive filler such as graphene should not introduce electrical conductivity to the thin-film product. Owing to the low graphene loading in the composites studied here, which is well below the percolation threshold, no electrical conductivity is observed in the thin-film composite samples tested here.

## 4 Conclusions

In conclusion, we have developed two varieties of water-based graphene/elastomer composite thin-films, which are 10s of microns thick. In the case of wPU, the graphene was incorporated as GO followed by in-situ reduction by ascorbic acid, whereas in the case of NRL, the graphene was first reduced to rGO and then added to the NRL so as to not disturb the stability of the latex emulsion. The graphene was well-dispersed as monolayers within the composites. In both composites, the elastic modulus was improved by 50% by the addition of just 0.1 wt % of large rGO flakes, while the elongation to failure was either maintained or improved. This is critical; as summarised in Ref. [12], the addition of graphitic fillers to elastomers invariably results in a loss of elasticity, either a lowered ultimate tensile strength or ultimate elongation to failure. The graphene loading was below the percolation threshold so the composites remain electrically insulating.

## Figures and Tables

**Table 1:** Mechanical properties of graphene/wPU composites.

Elongation (%)	Control wPU	0.05 wt% rGO	0.1 wt% rGO	0.2 wt% rGO
100	1.346	1.79993	2.01208	2.26822
200	1.77692	2.34687	2.622	2.85584
300	2.28896	3.1159	3.34462	3.52965
400	3.05701	4.13686	4.33906	4.47376
500	4.22557	5.6379	5.8506	5.83704
600	6.0025	8.30244	8.43945	8.1601
700	9.93656	–	13.81825	12.50067
Ultimate tensile strength (MPa)	12.81276	12.48957	17.12199	21.22293
Ultimate elongation (%)	744.93671	666.10127	748.63291	791.41772

**Table 2:** Mechanical properties of graphene/NRL composites.

Elongation (%)	Control NRL	Large GO	Large rGO	Small rGO
100	1.09542	1.03909	0.98902	1.05385
200	1.34562	1.36916	1.26583	1.40054
300	1.59699	1.60178	1.52759	1.71925
400	1.80664	1.8823	1.81379	2.06342
500	2.11442	2.2264	2.16141	2.50604
600	2.54785	2.60072	2.59132	3.00809
700	3.08992	3.18867	3.14805	3.67178
800	3.95925	4.14904	4.05382	4.65629
900	5.32649	5.67756	5.51543	6.02965
1000	7.08207	7.8023	7.63724	8.00367
1100	–	10.25085	9.34941	–
Ultimate tensile strength (MPa)	9.35309	12.68234	10.87765	8.19356
Ultimate elongation (%)	1088.596	1169.046	1129.055	1002.916
