# Supplementary material for: Graphene and water-based elastomers thin-film composites by dip-moulding
Source: Carbon N Y. 2016 Sep;106:228–32. doi: 10.1016/j.carbon.2016.05.032 (PMC4913555; doi:10.1016/j.carbon.2016.05.032)
Supplement: Supplementary file 1 [file mmc1.docx]

Graphene and water-based elastomers thin-film composites by dip-moulding – Supplementary data

Maria Iliut^a^, Claudio Silva^a^, Scott Herrick^b^, Mark McGlothlin^b^, Aravind Vijayaraghavan^a,^^[[1]](#footnote-1)^

*^a^ School of Materials and National Graphene Institute, University of Manchester, Manchester M13 9PL, UK
^b^ Apex Medical Technologies, San Diego, CA 92121, USA*


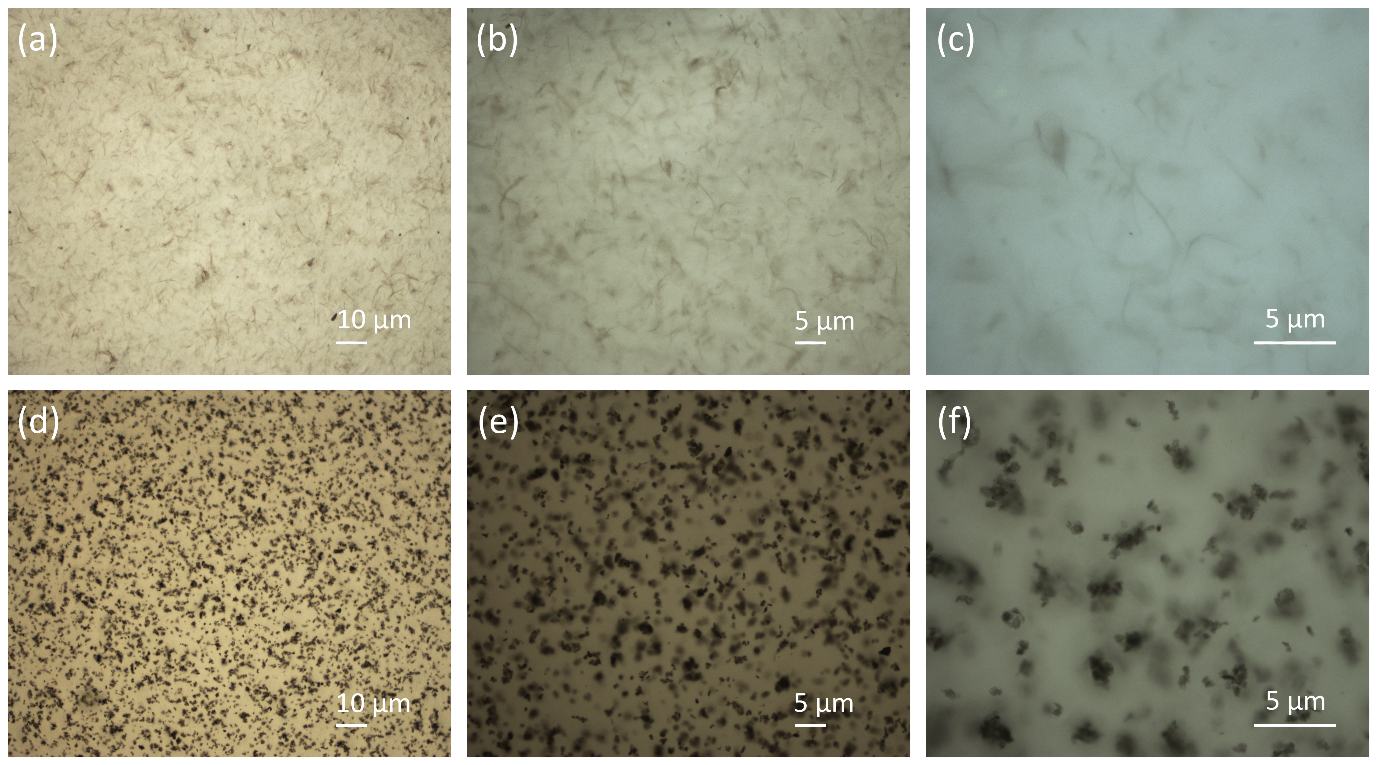


**Figure S1.** Optical micrographs of (a – c) **(a – c)** large and **(d – f)** small rGO flakes in NRL matrix at different magnifications.


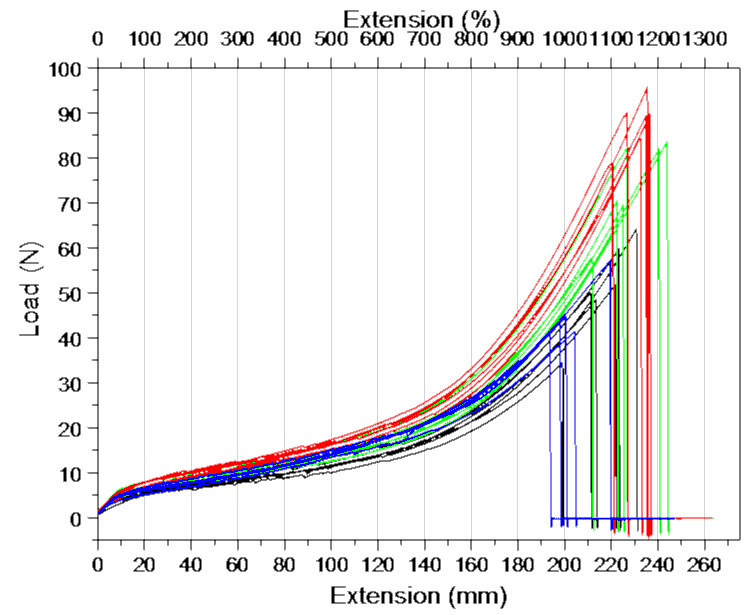


**Figure S2.** Raw load-displacement curves obtained for pure NRL (in black) and graphene/NRL composites as follows – NRL with big GO in red, with big rGO in green and with small rGO in blue.


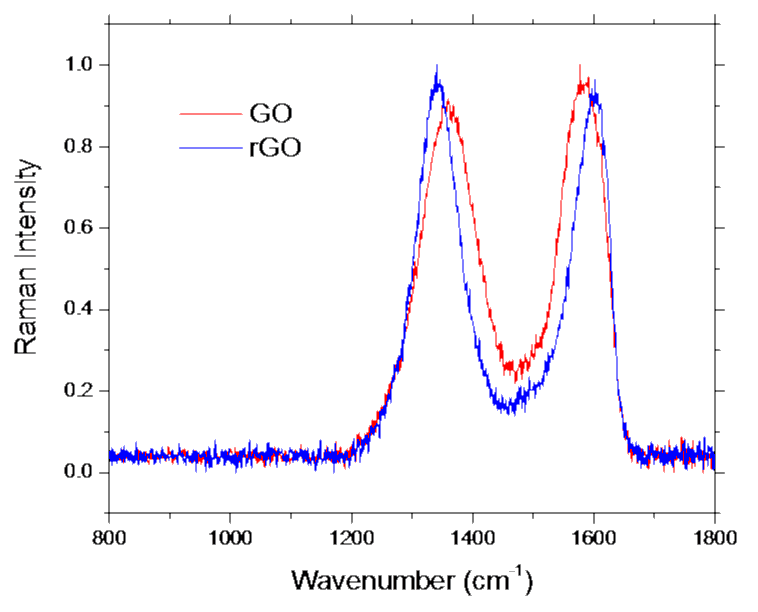

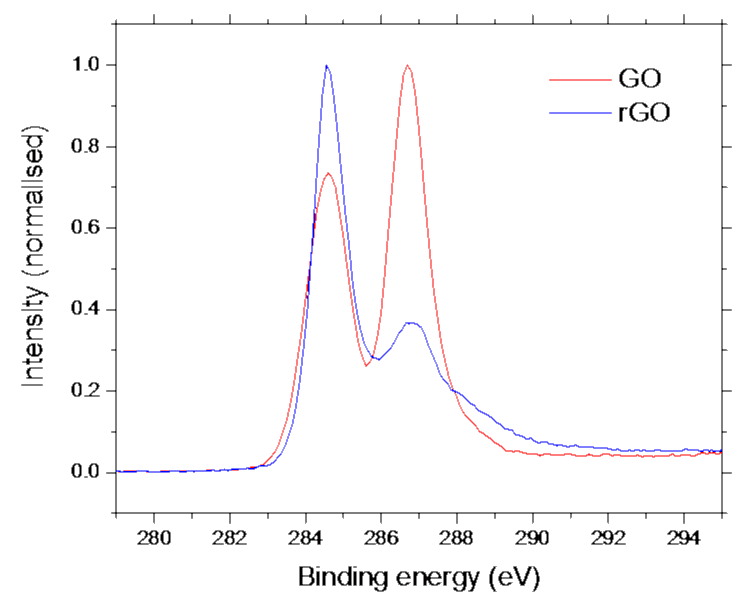


**Figure S3.** For GO and rGO used in the elastomer composites in this work, **a.** Raman spectra of GO and rGO showing higher D/G ratio and wider D/G spacing after reduction and **b.** XPS spectra (C1S peak) showing reduced C-O type peaks and higher C-C type peaks.

1. Corresponding author email: aravind@manchester.ac.uk [↑](#footnote-ref-1)
